# The role of intellectual property rights on access to medicines in the WHO African region: 25 years after the TRIPS agreement

**DOI:** 10.1186/s12889-021-10374-y

**Published:** 2021-03-11

**Authors:** Marion Motari, Jean-Baptiste Nikiema, Ossy M. J. Kasilo, Stanislav Kniazkov, Andre Loua, Aissatou Sougou, Prosper Tumusiime

**Affiliations:** Adjunct Faculty, Daystar University School of Law, Nairobi, Kenya

**Keywords:** Access to medicinal products, Intellectual property, TRIPS flexibilities, WHO, African region

## Abstract

**Background:**

It is now 25 years since the adoption of the Agreement on Trade-Related Aspects of Intellectual Property Rights (TRIPS) and the same concerns raised during its negotiations such as high prices of medicines, market exclusivity and delayed market entry for generics remain relevant as highlighted recently by the Ebola and COVID-19 pandemics. The World Health Organization’s (WHO) mandate to work on the interface between intellectual property, innovation and access to medicine has been continually reinforced and extended to include providing support to countries on the implementation of TRIPS flexibilities in collaboration with stakeholders. This study analyses the role of intellectual property on access to medicines in the African Region.

**Methods:**

We analyze patent data from the African Regional Intellectual Property Organization (ARIPO) and Organisation Africaine de la Propriété Intellectuelle (OAPI) to provide a situational analysis of patenting activity and trends. We also review legislation to assess how TRIPS flexibilities are implemented in countries.

**Results:**

Patenting was low for African countries. Only South Africa and Cameroon appeared in the list of top ten originator countries for ARIPO and OAPI respectively. Main diseases covered by African patents were HIV/AIDS, cardiovascular diseases, cancers and tumors. Majority countries have legislation allowing for compulsory licensing and parallel importation of medicines, while the least legislated flexibilities were explicit exemption of pharmaceutical products from patentable subject matter, new or second use of patented pharmaceutical products, imposition of limits to patent term extension and test data protection. Thirty-nine countries have applied TRIPS flexibilities, with the most common being compulsory licensing and least developed country transition provisions.

**Conclusions:**

Opportunities exist for WHO to work with ARIPO and OAPI to support countries in reviewing their legislation to be more responsive to public health needs.

## Background

The adoption of the Agreement on Trade-Related Aspects of Intellectual Property Rights [1] (TRIPS Agreement) in 1994 by Member States of the World Trade Organization (WTO) was a watershed event, which gave rise to a new global intellectual property protection (IPP) regime with significant effects on access to medicines. Some of these effects include high prices of medicinal products [2], prevention of local manufacture of generic products through reverse engineering of patented products, importation of cheaper medicinal products from off-patent countries or under licensing agreements and delayed market entry for generic products.

The potential impact of the TRIPS Agreement on access to medicines in developing and least developed countries, has caused debate with some commentators arguing that IPP makes it possible for pharmaceutical companies to recoup their Research and Development (R&D) costs and hence act as an incentive for investment in biopharmaceutical research [3] and innovation [4]. However, this incentive structure has failed to spur R&D investments for diseases that predominantly affect people living in countries with a high prevalence of neglected diseases [5], leading to the emergence of alternative product development partnership (PDP) models such as the Drugs for Neglected Diseases Initiative (DNDi) [4], proposals for a global R&D treaty,^1^ and the promotion of public health interests by using existing TRIPS flexibilities [6] or through revisions to the TRIPS Agreement.^2^

Although this paper focuses on the role of intellectual property rights on access to medicines, it is recognized that limited access to medicines in countries of the World Health Organization (WHO) African Region^3^ is a multidimensional problem. It is affected by other factors such as lack of public financing for health care and over-reliance on out of pocket expenditur e[7], fragile logistics, storage challenges and high transport and distribution costs [2] and inadequate or inappropriate medicines regulatory frameworks [8]. These factors are further exacerbated by insufficient scientific, technological and local manufacturing capabilities in the Region [9].

The occurrence of public health emergencies of global concern such as Ebola [10] and COVID19^4^ [11], have served to highlight further the tensions between IPP and access to medicines [12]. For example, in March 2020, Gilead Sciences, the makers of remdesivir which is a drug initially studied in clinical trials for Ebola Virus Disease (EVD) and has received US FDA emergency use authorization^5^ for the treatment of adults and children hospitalized with severe COVID-19 disease, made an application for orphan drug status for the drug, which has since been rescinded.^6^ Orphan drug designation for remdesivir would have granted Gilead Sciences 7 years of market exclusivity in addition to the standard 20 years of patent protection guaranteed by the TRIPS Agreement^7^ and other benefits such as tax credits of up to 50% of qualified clinical development spending, exemption from certain FDA fees and access to special FDA technical advice [13]. Gilead Sciences has since signed non-exclusive license agreements with pharmaceutical manufacturers in Egypt, India and Pakistan for the supply of remdesivir in 127 low and middle-income countries,^8^ which include all countries of the WHO African Region.

### WHO, intellectual property and access to medicines

The earliest articulation of WHO’s mandate to work on the interface between access to medical products, R&D in rare and tropical diseases, and trade can be traced back to 1996, in a World Health Assembly (WHA) resolution on the Revised Drug Strategy^9^ which requested the WHO Director-General (DG) to support Member States in their efforts to improve access to essential drugs; to encourage the promotion of R&D of drugs for rare and tropical diseases; and to report on the impact of WTO concerning national drug and essential medicines policies and make recommendations for collaboration between WTO and WHO as appropriate. This mandate has been continually reinforced through subsequent assembly resolutions.^10^ It has been extended over time to include upon request, providing technical and policy support to Member States, on formulating coherent trade and health polices and the implementation of TRIPS flexibilities in collaboration with other relevant international organizations.

In 2003, WHO member states agreed through resolution WHA56.27 to establish a Commission on Intellectual Property Rights, Innovation and Public Health (CIPIH) which recommended^11^ that “WHO should develop a global plan of action to secure enhanced and sustainable funding for developing and making accessible products to address diseases that disproportionately affect developing countries” and “ … continue to monitor from a public health perspective, the impact of intellectual property rights … on the development of new products as well as access to medicines and other health care products in developing countries”. These recommendations led to the adoption^12^ of the Global Strategy and Plan of Action on Public Health, Innovation and Intellectual Property (GSPOA-PHI) in 2009 and in the same year the WHO-WIPO-WTO trilateral cooperation, which is an interagency collaboration on public health, intellectual property and trade was commenced.

Most recent are assembly decisions WHA 71(8) of 2018 on ‘Addressing the global shortage of, and access to, medicines and vaccines’, which requested the DG to “elaborate a roadmap report, in consultation with Member States, outlining the programming of WHO’s work on access to medicines and vaccines including activities, actions and deliverables for the period 2019 – 2023; and WHA71(9) of 2018 on the ‘Global strategy and plan of action on public health, innovation and intellectual property (GSPOA-PHI): overall programme review’, which requested the DG to “implement the recommendations addressed to the Secretariat … in an implementation plan, consistent with the global strategy and plan of action on public health, innovation and intellectual property”. Additionally, in 2019 resolution WHA72.8 on ‘Improving the transparency of markets for medicines, vaccines, and other health products’, requested the DG to “continue supporting existing efforts to determine patent status of health products and promote publicly available user-friendly patent status information databases for public health actors, in line with the GSPOA-PHI and to work with other relevant international organizations and stakeholders to improve international cooperation, avoid duplication of work, and promote relevant initiatives”.

### Technical assistance for the implementation of TRIPS and the role of WIPO

Within the framework of the WHO-WIPO-WTO trilateral cooperation, the 3 agencies work collaboratively to each fulfill their respective mandates without duplicating efforts and within existing resource constraints. By virtue of the 1995 Agreement^13^ between WIPO and WTO, and recommendations^14^ of the WIPO Development Agenda (2007), WIPO plays an important role in providing developing countries with technical assistance to implement the TRIPS Agreement. Recommendation 1 states that “WIPO technical assistance shall be development-oriented, demand-driven and transparent, taking into account the priorities and the special needs of developing countries, especially LDCs”. An analysis of information provided on the WIPO website^15^ indicates that 24 countries^16^ of the WHO African Region have received technical assistance^17^ specific to the development of national IP strategies, policies and/or for legislative assistance. Based on the same information, Gabon, Ghana and South Sudan have not received any form of WIPO technical assistance.

### Aims of the study

The WHO commitments highlighted above and the fact that it is 25 years since the adoption of the TRIPS Agreement provide a good backdrop for reviewing the status of intellectual property rights and the use of TRIPS flexibilities in WHO African Region Member States. The study presents a situational analysis of patenting activity and trends at the African Regional Intellectual Property Organization^18^ (ARIPO) and the Organisation Africaine de la Proprieté Intellectuelle^19^ (OAPI), the two African regional patent offices. It reviews the intellectual property regulation and governance landscape affecting countries of the WHO African Region, including regimes established by ARIPO and OAPI. Finally, the study provides an assessment of the Region’s preparedness to respond to public health emergencies by analyzing how countries have implemented available TRIPS flexibilities in national legislation, including the disincentives and challenges experienced. The findings of the study provide a baseline for WHO’s work towards the implementation of the Health Assembly’s decision WHA 71(8) and resolution WHA72.8 mentioned above.

## Methods

### Patenting trends

The patent data used in this study was obtained from the ARIPO and OAPI offices in April 2019. Face-to-face meetings were organized with officers of the two organizations to explain the objectives of the study and to clarify that the data required was in the areas/fields that can potentially be applied in medical inventions using the World Intellectual Property Organization (WIPO) international patent classification (IPC) codes. Table 2 (see Appendix 1) presents the codes for patents falling within the scope of this study.

Officers of both patent offices conducted a data search using parameters defined and provided the requisite data as downloaded files in MS Excel. The total number of health-related patents registered in ARIPO and OAPI databases as of April 2019 was 3458 and 2811 respectively. This study only reviewed and analysed patents that had been granted within the past 20 years. Consequently, the patents analyzed were 960 (28%) for ARIPO and 2274 (81%) for OAPI. In order to identify the specific disease(s) a patent can be associated with, we used the patent short title, and in instances where the short title did not mention the disease, the patent abstract was consulted. Those that did not mention a specific disease were left out of the analysis. In doing the analysis the diseases covered by the patents were clustered in broad categories namely: inflammatory diseases, cancers, tumors and abnormal cell proliferation, cardiovascular diseases, viral infections, neurodegenerative diseases, diabetes and diabetes-related conditions, infections, pain, HIV/AIDS, tuberculosis, malaria, mental disorders, nervous system diseases, digestive system diseases, weight related disorders, lung diseases, eye diseases and vaccines.

### Application of TRIPS flexibilities

This study conceptualizes flexibilities as understood within the context of the TRIPS Agreement, its amending Protocol and the Doha Declaration on the TRIPS Agreement on Public Health (Doha Declaration).^20^ We define TRIPS flexibilities through the lens of Articles 1.1^21^ and 8.1,^22^ which provide policy space for countries to implement the TRIPS Agreement in a manner appropriate and responsive to their contexts, as different options which consider national interests and can be transposed into national law.

The study conducted a desk review of available patent laws and policy frameworks^23^ of WHO African countries with the aim of analyzing whether or not they have implemented TRIPS flexibilities within their national legal frameworks. To supplement this analysis, the study used data from The TRIPS Flexibilities Database, found on http://tripsflexibilities.medicineslawandpolicy.org/, which is a searchable publicly accessible database maintained by the Medicines Law and Policy group.^24^ The database contains records of instances when national authorities of WTO member countries have invoked the application of a TRIPS flexibility for public health reasons since 2001 to 2020. Finally, the study analysed information elicited from an online questionnaire administered by WIPO, and available on https://www.wipo.int/scp/en/exceptions/ to identify the most often cited challenges by respondent WHO African countries,^25^ in implementing TRIPS flexibilities.

The TRIPS Agreement uses the term flexibility in paragraph 6 of the preamble and in Article 66.1 in the context of the need for least developed countries (LDC) to create a viable technological base and their readiness to implement it. Paragraph 4^26^ of the Doha Declaration reaffirms the right of WTO members to use full provisions of the TRIPS Agreements and provides a contextual basis for understanding flexibilities while Paragraph 5 clarifies that they include;

- applying customary rules of interpretation of public international law so that all provisions of the TRIPS Agreement are read in light of the object and purpose as expressed in its objectives (Article 7) and principles (Article 8);
- the right of each member to grant compulsory licenses and the freedom to determine the grounds for granting such licenses;
- the right of each member to determine what constitutes a national emergency or other circumstances of extreme urgency, it being understood that public health crises include those relating to HIV/AIDS, tuberculosis, malaria and other epidemics; and
- the freedom of each member to establish its exhaustion of IPR regime without challenge subject to the national treatment (Article 3) and most-favored-nation treatment (Article 4) provisions.

Given that Paragraph 5 does not provide an exhaustive enumeration of flexibilities, the study highlights a number of applicable TRIPS flexibilities that African Region countries may evoke to enhance access to medicines. These flexibilities, which are discussed in detail in Table 3 (see Appendix 2), include:

i) An interpretation of Article 27.1 of the TRIPS Agreement in national legislation in a manner that excludes new uses, formulations, dosages or combinations of previously patented medicines from patentability criteria. This legislative measure coupled with the implementation of substantive patent examination procedures would serve to prevent frivolous patent applications, ever-greening and creation of patent thickets around one invention.
ii) Allowing limited exceptions to exclusive rights conferred by patents for purposes of scientific experimentation (research exception); and for facilitating regulatory and market entry approval (Bolar exception) in accordance with Article 30 of the TRIPS Agreement. The research exception makes it possible for countries to develop their local scientific and technological capacities and competencies to reverse engineer pharmaceutical products for generic production and for developing them further to better suit local conditions. On the other hand, the Bolar exception allows the use of a patented invention during the patent term without consent of the patent holder for purposes of developing information to obtain market approval and facilitates market entry by competitors immediately after the patent term expires hence ensuring early access to generic medicines.
iii) Compulsory licensing under Article 31 of the TRIPS Agreement, which allows for the exploitation of patented subject matter through government authorization without the patent holder’s consent, for reason of national emergency and public non-commercial use.
iv) Exhaustion of rights and parallel importation under Article 6 of the TRIPS Agreement and Paragraph 5 (d) of the Doha Declaration which make provision for importation and resale in a country without consent of the patent holder of a patented medicine put on the market of the exporting country by the patent holder or in a legal manner.
v) Patent term extension – Countries may consider disallowing/limiting patent term extension in national law for pharmaceutical products.
vi) Limits on test data protection – Article 39.3 of the TRIPS Agreement allows countries to determine how to protect test data in the public interest. This provision demands protection from unfair commercial use and does not demand data exclusivity. Countries may therefore incorporate in domestic legislation the right of regulatory authorities to rely on available data to assess new drugs for market entry.
vii) Least Developed Countries (LDCs) transition periods – These include the TRIPS Council decision^27^ for the extension of the transition period for LDCs under Article 66.1 of the TRIPS Agreement to 1 July 2021; the TRIPS Council decision^28^ and TRIPS General Council decision^29^ stating that LDC member states are not obliged to protect pharmaceutical patents, or to provide means for filing patents and provide exclusive marketing rights for pharmaceutical products until 1 January 2033.
viii) Creation of patent opposition (pre and post patent grant), as required by Article 62.4 of the TRIPS Agreement, to serve as an additional administrative layer of patents review to prevent the grant of invalid patents.

## Results

### Patenting trends

**Fig. 1:**
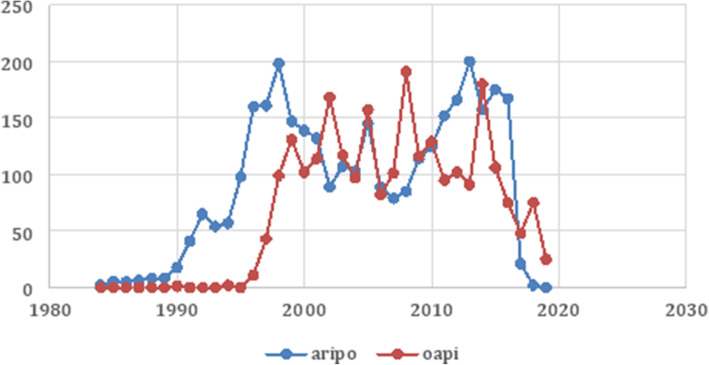
Health-related patent application trends at ARIPO and OAPI

Figure 1 below depicts health-related patent application trends at ARIPO and OAPI. Data shows that there was a sharp increase in patent applications at both offices from 1994 to 1999, which started to decline in the period 1999–2000. This trend matches that reported in a study on inventions and patenting in Africa [14]. The observed increase in patent applications in the late-1990s may have been caused by developments in molecular biology, genetics and genomics and their application in the biopharmaceutical sector.^30^

According to the 2018 Report on the State of Health in the WHO African Region [15], the main five causes of morbidity and mortality in the Region, are lower respiratory infections, HIV/AIDS, diarrheal diseases, malaria and tuberculosis. Other top causes of morbidity and mortality include stroke and ischemic heart diseases, preterm birth complications, birth asphyxia and congenital anomalies.

An analysis of the top 10 diseases covered by patents granted at ARIPO and OAPI corresponds to a certain extent, with the top 10 causes of morbidity as illustrated by Figs. 2 and 3 below. In both repositories the three top categories of diseases covered by granted patents were inflammatory diseases; cancers, tumors and abnormal cell proliferation; and cardiovascular diseases. Within the inflammatory diseases category, there were patents for lower respiratory diseases such as chronic obstructive pulmonary disease and asthma. HIV/AIDS, which was the second cause for morbidity and mortality, was 7th among the top 10 ARIPO patents and 10th for OAPI. Stroke and ischemic heart diseases which ranked 4th and 5th as causes of mortality respectively, fall within the broad category of patents covering cardiovascular diseases, which ranked 3rd on the list of top 10 diseases covered by patents at ARIPO and OAPI.

**Figure:**
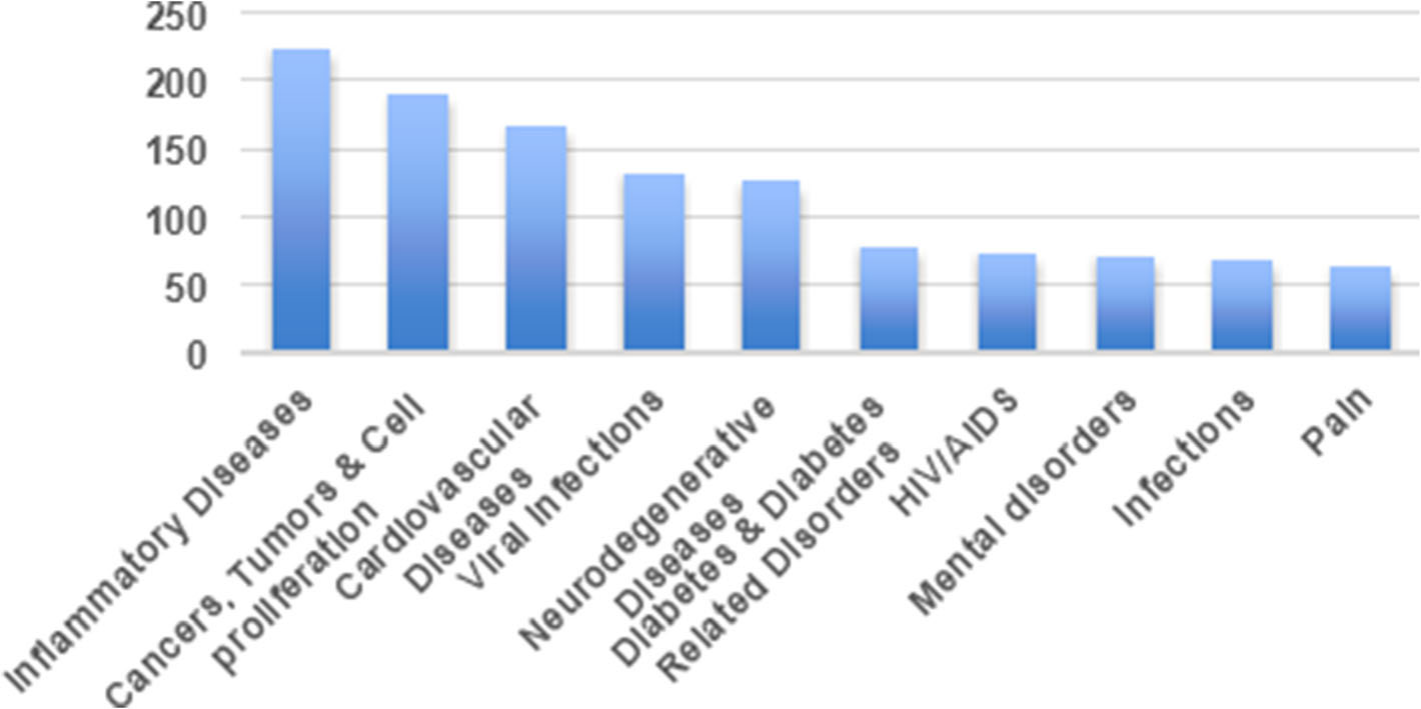
**Fig. 2** Top 10 diseases covered by patents granted by ARIPO

**Figure:**
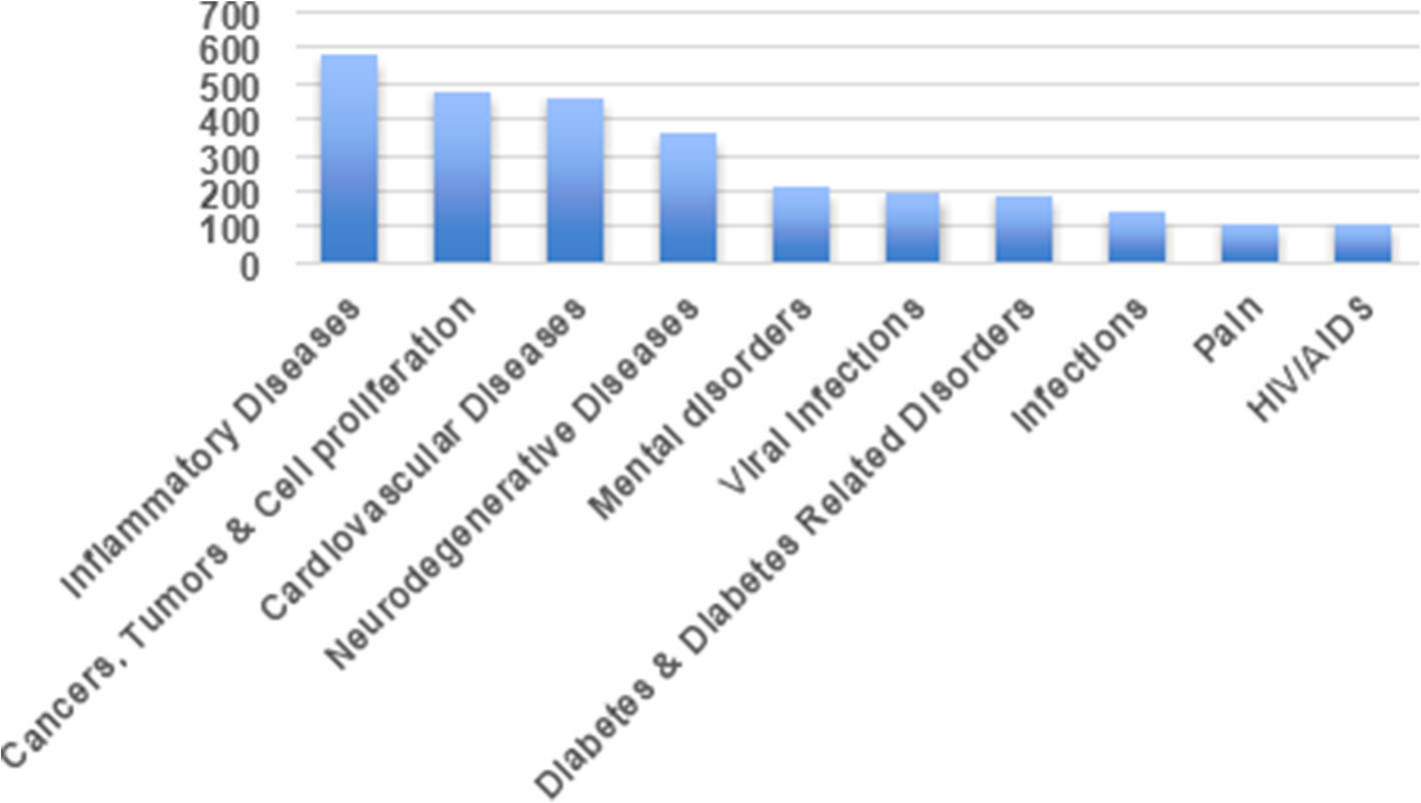
**Fig. 3** Top 10 diseases covered by patents granted by OAPI

Diarrheal diseases, tuberculosis and malaria, which are among top causes of mortality and morbidity did not appear among the top diseases covered by patents in the Region. There were only 23 TB patents at ARIPO and 25 at OAPI, while there were only 37 malaria patents at ARIPO and 47 at OAPI.

The top 10 categories of diseases covered by patents taken at ARIPO and OAPI were the same, the only difference being in the ranking. Another interesting finding, whose implications merit further interrogation, is the ranking of cancer-related patents, which was 2nd at both patent offices.

An analysis of the countries of origin of health-related patents at ARIPO and OAPI shows that the top 10 sources of patents at ARIPO were USA, Great Britain, the European Patent Office, France, India, China, South Africa, Germany, Italy and Denmark. South Africa was the only African country in the top 10 list. The top 10 sources of patents at OAPI were USA, France, Great Britain, Germany, India, Belgium, Japan, Cameroon, Switzerland and Ireland. Cameroon was the only African country in the top 10 list. The African Region countries that had health-related patents at ARIPO were Kenya, Mauritius, Namibia, Zambia, and Zimbabwe and Egypt from the Eastern Mediterranean Region (EMRO) of the WHO. While in OAPI the African Region countries were Burkina Faso, Benin, Central African Republic, Congo Republic, Cote d’Ivoire, Cameroon, Guinea, Mali, Mauritius, Namibia, Nigeria, Senegal, Togo, South Africa and Egypt and Morocco from EMRO.

An analysis of the African country patents shows that the top three disease categories covered are HIV/AIDS, cardiovascular diseases, and cancer and tumors. Table 1 below provides a summary of the main diseases covered by African country patents.

**Table 1:** Summary of main diseases covered by African country patents

Disease	Number of mentions in patents	Disease	Number of mentions in patents
HIV/AIDS	20	Tuberculosis	5
Cardiovascular diseases	18	Bacterial infection	5
Cancer and Tumors	14	Antimicrobial	4
Diabetes	14	Antiviral	4
Malaria	13	Ulcers	4
Skin	10	Hemorrhoids	4

### The intellectual property regulation and governance landscape in the WHO African region

Countries of the WHO African Region operate within a multi-layered IP regulation and governance landscape. The Paris Convention for the Protection of Industrial Property (1883) and the TRIPS Agreement (1994) regulate the global IP framework within which African Region countries operate. Most countries (40 out of the 47) in the Region are members of the World Trade Organization (WTO) and are Parties to the Agreement on Trade Related Aspects of Intellectual Property Rights (TRIPS Agreement), which provides a minimum standard for the regulation of intellectual property. Article 1.1 of the TRIPS Agreement states that signatory Members States may, but shall not be obliged to, implement in their law more extensive protection than is required by TRIPS, making it open for them to determine the appropriate level of implementing it within their own legal system and practice. At the time of writing this article Algeria, Comoros, Equatorial Guinea, Eritrea, Ethiopia, Sao Tome and Principe, and South Sudan were not members of the WTO. All countries with the exception of Eritrea had commenced accession discussions leading to eventual membership.

At the Regional level, countries are either signatories to the Harare Protocol on Patents and Industrial Design (1982), which is administered by ARIPO or the Bangui Agreement (1977) administered by OAPI.

### The African regional intellectual property office (ARIPO) system and TRIPS flexibilities

The African Regional Intellectual Property Office (ARIPO) was established by the Lusaka Agreement in 1976; and has 20 member states, 18^31^ of whom are members of the WHO African Region. The objective of ARIPO is to promote the harmonization and development of intellectual property laws appropriate to the needs of its members, fostering the establishment of a close relationship between its members on intellectual property matters and establishing such common services or organs as may be necessary or desirable for the co-ordination, harmonization and development of intellectual property activities affecting its members.

The Harare Protocol on Patents and Industrial Designs (Harare Protocol) was adopted in 1982 and came into force in 1984. There are 18 contracting parties to the Harare Protocol namely Botswana, Eswatini, Gambia, Ghana, Kenya, Lesotho, Liberia, Malawi, Mozambique, Namibia, Rwanda, Sao Tome and Principe, Sierra Leone, Sudan, Tanzania, Uganda, Zambia and Zimbabwe. Of these, Gambia, Lesotho, Liberia, Malawi, Mozambique, Rwanda, Sao Tome & Principe, Sierra Leone, Uganda, Tanzania and Zambia are classified as LDCs.

Under the Harare Protocol, an application for the grant of a patent is made with any Contracting Party or directly with ARIPO, in which the applicant designates any one of the Contracting Parties in which they wish the invention to be accorded protection. The Protocol establishes a regional mechanism that administers the filing, examination and grant of patents, in all fields of technology, including the pharmaceutical field (section 3.10.a). A review of how the TRIPS flexibilities identified in the methods section above are implemented by the Harare Protocol shows that patentability criteria extends to cover pharmaceutical products under Rule 10 of the Regulations to the Harare Protocol. Additionally, Rule 7 (3) on drafting patent claims implies that ARIPO allows claims relating to second uses of known and already patented pharmaceutical products, which is likely to encourage frivolous patent applications and ever-greening.

Rule 3.3 of the Regulations to the Harare Protocol makes provision for ARIPO to, upon request, undertake or arrange for the substantive examination of a patent application. According to one of the ARIPO officials interviewed for this study, substantive examination is ordinarily conducted at ARIPO. However, ARIPO has bilateral agreements with the European Patent Office (EPO), the Australian, German and Swedish patent offices as well as with WIPO, for the conduct of substantive examination in technical fields or for complex inventions that ARIPO may not have adequate examination capacity.^32^ A study conducted in 2014 [16] revealed that ARIPO had only 6 patent examiners at the time, which is a grossly low number of examiners. Rule 19bis of the Regulations to the Harare Protocol provides for the publication of patent applications as soon as possible after the expiry of 18 months from the date of filing or from priority date, and does not make provision for opposition of patents granted by ARIPO.

### The organisation Africaine De La Propriété Intellectuelle (OAPI) system and TRIPS flexibilities

The Organisation Africaine De La Propriété Intellectuelle (OAPI) was created in 1977 by the Bangui Agreement and has the objective of implementing and applying common administrative procedures deriving from a uniform system for the protection of industrial property, providing services related to industrial property, and promoting the economic development of Member States by means of effective protection of intellectual property and related rights, according to Article 2.1 of the Agreement. According to Articles 2 (2), 8 (1) and 8 (2) of the Bangui Agreement OAPI serves as both a national and central patent documentation body for all its member countries and for Member States party to the Patent Cooperation Treaty (PCT). In this case, OAPI serves as the national, designated, elected, and/or receiving office within the context of Article 2^33^ of the PCT, and the Bangui Agreement is the law governing industrial property rights in each of its member states. This being the case, patent applications at OAPI are designated for protection in all OAPI member countries.

The countries signatory to the Bangui Agreement are Benin, Burkina Faso, Cameroon, Central African Republic, Chad, Congo Republic, Cote d’Ivoire, Gabon, Guinea, Guinea-Bissau, Mali, Mauritania, Niger, Senegal and Togo. Out of the 15 Member States, only 4 countries are not LDCs namely Cameroon, Congo Republic, Cote d’Ivoire, and Gabon.

According to the Bangui Agreement a patentable invention is defined as a product or process that is new, involves an inventive step and is industrially applicable. This broad definition of patentability criteria is construed to cover both pharmaceutical products and processes. In accordance with provisions of Article 20 of the Annex to the Bangui Agreement, OAPI is a formality and not a substantive examination office. Therefore all patent applications that meet the formality examination requirements namely, Articles 6,^34^ 14.1,^35^ and 15^36^ of the Annex to the Agreement, are granted under Article 22. The Bangui Agreement just like the Harare Protocol does not make provision for a patent opposition system that would serve to prevent granting of invalid and or frivolous patents.

### Implementation and application of TRIPS flexibilities by WHO African region countries

Table 4 (see Appendix 3) below presents a summary how the TRIPS flexibilities identified in the methods section and discussed in Table 3 (on Appendix 2 below) have been enacted into law by African Region countries. The summary is based on a review of all WHO African countries’ patent legislation and IP policies as accessed through the WIPO Lex depository.^37^

The analysis shows that 3 countries, namely Namibia,^38^ Rwanda^39^ and Zambia^40^ have specific legislation on patentability of pharmaceutical products based on what constitutes novelty to limit ever-greening of patents. These countries have explicit legislation against new or second use patents of already patented pharmaceutical products. Twenty one (21) countries^41^ make provision for limited exceptions to exclusive patent rights for purposes of research and scientific experimentation (research exception), while twelve^42^ allow for the use of patented knowledge during the patent term for purposes of developing information necessary for attaining regulatory and market-entry approval (bolar exception). The Bangui Agreement as currently enforced, does not make provision for research and bolar exceptions for signatory member states.

The most commonly legislated flexibilities are compulsory licensing and parallel importation where 45 out of the 47 (95%) countries have enacted legislation to allow for compulsory licensing, and 40 (85%) for exhaustion of rights and parallel importation. The 2 countries that do not legislation on compulsory licensing were Eritrea and Madagascar and the 7 without legislation on parallel importation are Angola, Cabo Verde, Comoros, Ethiopia, Eritrea, DRC and Malawi.

The least commonly legislated flexibilities were those imposing limits to patent term extensions and on test data protection. Only Angola^43^ and Zimbabwe^44^ were found to have legislative provisions that could be used to limit pharmaceutical product patent term extensions, while only Uganda has legislation which allows the medicines regulatory authority to rely on available data to assess new generic drugs for market entry.

Out of the 30 WHO African Region member states who are classified as LDCs^45^ and qualify for pharmaceutical product patent waivers, until 1 January 2033, on the mailbox provision and exclusive marketing rights for pharmaceuticals relating to Articles 70.8 and 70.9 of the TRIPS Agreement, only Angola,^46^ Madagascar,^47^ Liberia,^48^ Rwanda^49^ and Uganda^50^ have explicitly excluded pharmaceutical products from patentability criteria in their national laws. Burundi’s Industrial Property law^51^ states that this exemption was valid until 1 January 2016. Least developed countries signatory to the Bangui Agreement and members of OAPI do not exempt pharmaceutical products from patentable subject matter. Algeria, Angola, Eritrea, Ethiopia, Madagascar, Nigeria, Sierra Leone and all OAPI countries were found not to have any patent opposition procedures (whether pre and/or post grant) in place.

An analysis of the TRIPS flexibilities database^52^ summarized in Table 5 (see Appendix 4) below shows that 39 out of the 47 African Region Countries have used one or more TRIPS flexibility at one time or another. African Region countries that were not recorded on the TRIPS flexibilities database as having applied any flexibility are Algeria, Botswana, Madagascar, Mali, Mauritius, Namibia, Nigeria, and Seychelles. Majority of the applications were for sourcing treatments for HIV/AIDS, except in the cases of Angola, Cape Verde, Chad, Gambia, Kenya, Lesotho, Malawi, Niger, Rwanda, South Sudan, Tanzania and Togo that applied flexibilities for sourcing all medicines.

The analysis shows that only three flexibilities are recorded in the database as having been applied, namely Article 31 of TRIPS which allows for compulsory licensing including for non-commercial use; paragraph 7 of the Doha Declaration on LDC country transition periods and paragraph 5 (d) of the Doha Declaration allowing for parallel importation. The most commonly applied flexibility is paragraph 7 of the Doha Declaration on transition provisions with 27 countries having applied it, followed by Article 31, allowing compulsory licensing, which has been applied by 16 countries. Parallel importation has only been used by Kenya once (in 2002) for the importation of generic medicines.

Some countries have applied flexibilities more than once; the highest being five times by Kenya, four times by Zimbabwe, and three times by Benin, Congo, Gabon, Ivory Coast, Mozambique, Togo and Zambia. Central African Republic, Chad, the Gambia, Guinea, Lesotho, Malawi, Niger, Rwanda and Sierra Leone have all applied flexibilities twice respectively. Guinea, Mozambique and Zambia adopted a mixed approach of using both Article 31 and paragraph 7, and Kenya both Article 31 and paragraph 5 at different times. In the case of Kenya, it did not execute 4 of its applications which were under Article 31 for HIV/AIDS medications. The pharmaceutical companies involved in these cases, GSK and Boehringer Ingelheim, entered into voluntary license agreements with a Kenyan manufacturing company Cosmos Ltd. The 5th application by Kenya, and the only one to be executed was under paragraph 5 and related to sourcing of generic drugs. Cameroon and South Africa too did not execute their flexibilities applications which were both under Article 31 and were both for HIV/AIDS drugs. The database does not record why Cameroon did not execute its application. In the case of South Africa, the concerned pharmaceutical companies GSK and Boehringer Ingelheim entered into a voluntary license with Cipla, an Indian manufacturing company.

An analysis of responses provided by twelve WHO African countries^53^ to an online questionnaire administered by WIPO,^54^ identifies a number of challenges that countries experience in applying TRIPS flexibilities to meet public health needs. The most often cited challenge in the survey was insufficiency or no local manufacturing capacity [17] to produce generic pharmaceutical products in relation to the use of compulsory licensing. This arose from the fact that Article 31 (f)^55^ and (h)^56^ of the TRIPS Agreement, before entry into force of Article 31*bis* in 2017*,* which made it impossible for countries with insufficient or no pharmaceutical manufacturing capacity to use compulsory licensing to access patented medicines. It was however noted from the survey that the considerable burden of proof on governments and potential users of the Article 31*bis* system^57^ remains to be a challenge. Another challenge identified from the WIPO survey and in literature [18, 19], is the risk of having counterfeit pharmaceutical products introduced into the market through parallel importation.

## Discussion

The WHO African Region is characterized by what could be termed as a collage of member states as far as IP regulation and governance is concerned. To begin with, we have countries such as Algeria, Comoros, Equatorial Guinea, Eritrea, Ethiopia, Sao Tome and Principe and South Sudan who are not yet signatories to the TRIPS Agreement and are exempt from any intellectual property protection requirements imposed by the Agreement that may hinder access to medicine. Out of the other 40 WHO African Region Member States, 21^58^ signatories to the TRIPS Agreement are unlikely to benefit from the LDC transition waivers under Article 66.1 for fully implementing the TRIPS Agreement until 1 July 2021; and on obligations under Article 70.8^59^ and 70.9,^60^ with respect to mailbox applications and provision of exclusive marketing rights of pharmaceutical products until 1 January 2033^61^ since they are signatories to the Harare Protocol or the Bangui Agreement (1977). The Harare Protocol and the Bangui Agreement require these Member States to attain a TRIPS – plus standard, where patentability criteria extends to pharmaceutical products and process, hence more onerous than the one required under TRIPS.

The IP frameworks imposed on ARIPO and OAPI Member States are inconsistent and misaligned with the TRIPS Agreement and are more onerous than the minimum standard provided by TRIPS. Areas of misalignment that have an impact on access to medicines in countries is the uniform treatment of LDC member states with non LDCs hence the lack of differentiation between obligations for LDCs and non LDC; the absence of capacity to conduct substantive patent examination to ensure that patentability criteria are met prior to granting patents, the absence of patent opposition procedures, failure to impose limits on pharmaceutical patent term extension and on test data protection to prohibit data exclusivity especially in the interest of public health.

In the years following the adoption of the TRIPS Agreement, developing countries experienced challenges in applying TRIPS flexibilities such as compulsory licensing (Article 31) and parallel importation (Article 6) of drugs in their bid to address the HIV/AIDS crisis that was facing most developing countries towards the end of the twentieth century. One such example in the African Region was South Africa, which enacted the Medicines and Related Substances Control Amendment Act 1997 that allowed for parallel importation and compulsory licensing of pharmaceuticals in the country. These amendments to the law led to a backlash from pharmaceutical companies and culminated into legal action against the South African government, which was later withdrawn [20, 21]. Over time and with a lot of public pressure on the pharmaceutical industry African countries have been able to put in place legislation allowing them to exploit some available TRIPS flexibilities to address public health needs. Though some positive strides have been made by WHO African Region countries, a lot more can be done by legislating for a wider range of flexibilities such a more rigorous application of the LDC transition waivers, adopting rigid patentability criteria which prohibits new or second use of already patented pharmaceutical products, limiting patent term extensions and limiting test data protection to facilitate faster entry of generic medicines into the market.

Compulsory licensing was a commonly evoked flexibility by countries, which in some instances was not executed, hence the conclusion that these may have served to encourage pharmaceutical companies to enter into voluntary licensing arrangements.

We observe that exhaustion of rights and parallel importation is not commonly applied in the Region, despite being one of the most legislated flexibility. This could be due to the fact that some countries have adopted a national exhaustion approach such, while those governed by the Bangui Agreement have precluded international exhaustion and restricts parallel importation to the regional exhaustion regime within OAPI countries. It could also be due to fears of proliferation of substandard and counterfeit medicines into the market.

## Conclusions

The experiences of the HIV/AIDS pandemic in the nineties, and most recently, the search for an effective treatment and vaccine for COVID-19 highlight the tensions between intellectual property rights and public health interests. There is evidence that learning has taken place from the lessons of the HIV/AIDS pandemic. Some of these lessons include the willingness by pharmaceutical companies such as Gilead Sciences to enter into voluntary licensing agreements with manufacturing companies in developing countries to serve less developed markets. The speed with which countries such as Canada, Germany, Chile and Ecuador have amended their respective patent laws to prohibit market exclusivities and to allow for compulsory licensing, should it become necessary, of COVID-19 medicinal products are examples of how TRIPS flexibilities can be deployed to address health needs.

The low levels of patenting activity by African Region countries calls for the need to develop and strengthen health innovation systems in the Region. This can be done through policies that support health research systems and a local incentive structure that focuses research on local health challenges. Other aspects of developing health innovation systems would include developing local scientific and biomedical research capacities and local manufacturing capabilities.

The findings of this study provide an opportunity for the WHO Regional Office for Africa to work closely with ARIPO and OAPI to develop and promote a Regional IP framework that is responsive to public health challenges of the Region; and to support countries in reviewing national IP laws taking into account available flexibilities especially the LDC transition waivers and those not commonly used in the Region such as research exception, regulatory review exception and patent term extension.

## Data Availability

The datasets generated and/or analysed during the current study are not publicly available due to the fact that they are held by ARIPO and OAPI patent depositories but are available from the corresponding author on reasonable request.

## Appendix 1

**Table 2:** IPC Codes of patents with potential application in the pharmaceutical sector

IPC Classification	Description
A61K	Preparations for medical purposes
A61P	Therapeutic activity of chemical compounds or medicinal preparation
A61B	Diagnosis - analyzing biological material covering processes for diagnostics.
A01N	Medicinal preparations (for pharmaceutical use) containing materials from mammals or birds
C07H	Sugars, derivatives thereof; nucleosides, nucleotides, nucleic acids with therapeutic activity further classified in subclass A61P, which covers DNA or RNA concerning genetic engineering, vectors or the isolation, preparation or purification
C12N	Micro-organisms or enzymes; compositions thereof; propagating, preserving or maintaining micro-organism, mutation or genetic engineering; culture media
C12P	Fermentation or enzyme using processes to synthesize a desired chemical compound or composition or to separate optical isomers from racemic mixture; particularly processes for producing enzymes, DNA or RNA concerning genetic engineering, vectors e.g. plasmids or their isolation.
C12Q	Measuring or testing processes involving enzymes or micro-organisms (immunoassays) compositions or test papers thereof; processes of preparing such composition; condition responsive control in microbiological or enzymological process.
C08B	Polysaccharides, derivatives thereof- those with therapeutic activity are further classified in subclass A61P.
C07F	Acyclic, carbocyclic, or heterocyclic compounds containing elements other than carbon, hydrogen, halogen, oxygen, nitrogen, sulfur, selenium or teleum. Therapeutic activity in A61P
C01G	Compounds containing metals and whose therapeutic activity is further classified under A61P
C07J	Steroids particularly those whose therapeutic activity is further classified in subclass A61P.
C08L	Compositions of macromolecular compounds which include pharmaceuticals under A61K
C07G	Organic chemistry/compounds of unknown constitution. Those with therapeutic activity further classified in subclass A61P
C07B	General methods of organic chemistry; apparatus therefore (preparation of carboxylic acid esters by telomerization)
C07C	Acyclic or carbocyclic compounds
C07D	Heterocyclic compounds. Those with therapeutic activity further classified in subclass A61P
C08G	Macromolecular compounds obtained otherwise than by reactions only involving carbon-to-carbon unsaturated bonds.
G01N	Investigation or analyzing materials by determining their chemical or physical properties.
C01B	Non-metallic elements; compounds thereof; whose therapeutic activity is further classified in A61P
C08F	Macromolecular compounds obtained by reactions only involving carbon-to-carbon unsaturated bonds; which include therapeutic activity under A61K, A61P

## Appendix 2

**Table 3:** Summary of available TRIPS flexibilities

Flexibility	Legal Basis	How and Why to Implement the Flexibility
Patentability criteria and exemption from patentability based on what constitutes novelty.	Article 27.1 of TRIPS does not specify what constitutes “new” or how the novelty requirement should be met. According to Para 4 of the Doha Declaration countries may interpret provisions of this Article in a manner that seeks to protect public health and ensure access to medicines. The TRIPS Agreement requires countries and jurisdictions to adopt and implement substantive and procedural examination procedures to ensure that patentability criteria are met prior to granting patents. Substantive examination of patent applications provides a higher degree of certainty as to the novelty and veracity of knowledge covered in a patent than that provided by a system that simply registers patent applications.	Countries may opt to interpret ‘novelty’ in domestic legislation in a manner that excludes new and second uses of medicines. This flexibility is applied to prevent frivolous patent applications from new uses, formulations, dosages or combinations of known or previously patented medicines, evergreening, or the creation of patent thickets around one invention.
Research exception	Article 30 of TRIPS states that “Members may provide *limited exceptions* to the exclusive rights conferred by a patent, provided that such exceptions do not unreasonably conflict with a normal exploitation of the patent and do not unreasonably prejudice the legitimate interests of the patent owner, taking into account of the legitimate interests of third parties.	The terms *limited exceptions* have been defined by the WTO Dispute Settlement Panel^a^ as “exception under which use of the patented product for scientific experimentation, during the term of the patent without consent is not infringement” Countries can therefore allow this exception in their national law in order to allow for the development of local scientific and technological knowledge and competencies to reverse engineer pharmaceutical products for generic production and for developing them further to better suit local conditions.
Regulatory review exception (Bolar exception)	This is also anchored in Article 30 of TRIPS.	This exception allows the use of a patented invention during the patented term without consent of the patent holder for purposes of developing information to obtain market approval. It allows generic producers to use patented knowledge for purposes of attaining regulatory approval for generic products before expiry of the patent(s), while the patent holder has the right to request, within specified conditions, an extension of the patent term to compensate for the delay in the drug regulatory and market approval process. This exception facilitates market entry by competitors immediately after the end of the patent term and is therefore an instrument that is specifically designed to ensure early access to generic medicines.
Compulsory licensing	Article 31 of TRIPS allows exploitation of patented subject matter through government authorization without the patent holder’s consent, for the supply of the domestic market of the member authorizing such license, for reason of national emergency and public non-commercial use. Paragraph 6 of the Doha Declaration and Article 31(bis) of TRIPS makes it possible, under specific conditions for compulsory licensing for export through regional trade areas (RTAs), which make it possible for bulk purchases and economies of scale. Para 5 (b) the Doha Declaration clarifies that each member has the right to grant compulsory licenses and the freedom to determine the grounds upon which such licenses are granted.	Article 31 of TRIPS stipulates conditions for the issuance of a compulsory license by a member state. Provisions of Article 31 (f) and (h) make it impossible for countries with insufficient or no manufacturing capacity to use this flexibility. Questions of market viability for a single national market made it unattractive to use this flexibility. Para.6 of the Doha Declaration sought a solution to situations where a foreign producer can supply patented pharmaceutical products, which are not available in a country with no sufficient manufacturing capacity. This has been addressed through Article 31(*bis*), which entered into force on 23 January 2017.
Exhaustion of rights and parallel importation	Article 6 of TRIPS states that “For the purpose of dispute settlement under this Agreement, subject to the provisions of Articles 3 and 4 nothing in this Agreement shall be used to address the issue of the exhaustion of intellectual property rights” Para 5(d) of the Doha Declaration reaffirms that countries are free to determine their own regimes for exhaustion of patent rights without challenge.	This provision allows importation and resale in a country without consent of the patent holder of a patented product put on the market of the exporting country by the patent holder or in a legal manner. International level of exhaustion provides the most flexibility, followed by regional and lastly national. It is a legal doctrine according to which an IP right holder cannot prevent further distribution or resale of goods after consenting to the first sale. In such a situation the right holder is considered to have exhausted its right over those goods.
Patent term extension	Article 33 of TRIPS provides that the patent term is 20 years from the filing date.	Countries may consider limiting patent term extension in national law for pharmaceutical products.
Limits on test data protection	Article 39.3 of TRIPS states that “Members, when requiring, as a condition of approving the marketing of pharmaceuticals … which utilize new chemical entities, the submission of undisclosed test or other data, the origination of which involves considerable effort shall protect such data against unfair commercial use. In addition, Members shall protect such data against disclosure, except where necessary to protect the public, or unless steps are taken to ensure that the data are protected against unfair commercial use.”	This provision allows countries to determine how to protect test data in the public interest. This provision does not demand data exclusivity, which has the potential of blocking entry of generic versions of patented medicines. Rather, it demands protection from unfair commercial use. TRIPS does not define unfair commercial use nor does it provide guidance on how protection can be achieved. Countries may therefore incorporate in domestic legislation the right of regulatory authorities to rely on available data to assess new drugs for market entry.
Transition periods for LDCs	Article 66.1 of TRIPS states that “In view of the special needs and requirements of least developed country Members, their economic, financial and administrative constraints, and their need for flexibility to create a viable technological base, such Members shall not be required to apply the provisions of this Agreement, other than Articles 3, 4 and 5, for the period of 10 years from the date of application as defined under paragraph 1 of Article 65. The Council of TRIPS shall, upon duly motivated request by a least-developed country Member in order to enable them to create a sound an viable technological base” Para7 of the Doha Declaration states that ‘… LDC will not be obliged, with respect to pharmaceutical products to implement or apply Sections 5 and 7 of Part II of the TRIPS Agreement or to enforce rights provided for under these Sections …’ TRIPS Council decision IP/C/64 of 2013 for the extension of transition period for LDCs under Article 66.1 to 1 July 2021. TRIPS Council decisions IP/C/73 and TRIPS General Council decision WT/L/971 stating that LDC members are not obliged to protect pharmaceutical patents or to provide means for filing patents and provide patent protection and exclusive marketing rights until January 2033.	In 2013 the TRIPS Council decided to extend the transition period for LDC members to fully comply with the TRIPS Agreement to 1 July 2021. LDC Members are exempt from the patentability requirement under Article 27.1 of TRIPS covering pharmaceutical products until 2033, also known as the pharmaceutical product transition period
Enactment of a patent opposition system	Under Article 62.4 of the TRIPS Agreement Member States are required to enact laws that provide procedures for the acquisition and maintenance of intellectual property rights as well as for administrative revocation, opposition and cancellation. These procedures are to be guided by principles of fairness and equity and should not be unnecessarily complicated or costly, or entail unreasonable time limits or unwarranted delays according to Article 41.2 of the TRIPS Agreement.	A patent opposition system could function pre or post patent grant and serves as an additional administrative layer of review that prevents the grant of invalid patents through the participation of third parties in the review process.

^a^In the Canada – Patent Protection of Pharmaceutical Products dispute, WT/DS114/R

## Appendix 3

**Table 4:** Implementation of available TRIPS Flexibilities by WHO African Region Countries

Flexibility	WHO African countries with relevant legislation	Observation
Patentability criteria and exemption from patentability based on what constitutes novelty.	Namibia, Rwanda and Zambia have specifically legislated against new or second use of already patented pharmaceutical products to stop “ever-greening” of patents	
Research exception	Algeria, Botswana, Burundi, Cabo Verde, Democratic Republic of Congo, Eswatini, Ethiopia, Ghana, Kenya, Lesotho, Liberia, Madagascar, Mauritius, Mozambique, Namibia, Rwanda, Sao Tome & Principe, Seychelles, Sierra Leone, Tanzania, Uganda,	OAPI countries –Benin, Burkina Faso, Cameroon, Central African Republic, Chad, Comoros, Congo, Cote d’Ivoire, Equatorial Guinea, Gabon, Guinea, Guinea Bissau, Mali, Mauritania, Niger, Senegal and Togo do not provide legislative exceptions for research purposes.
Regulatory review exception (Bolar exception)	Botswana, Cabo Verde, Kenya, Liberia, Namibia, Rwanda, Sao Tome & Principe, Seychelles, South Africa, Uganda, Zambia, Zimbabwe	OAPI countries –Benin, Burkina Faso, Cameroon, Central African Republic, Chad, Comoros, Congo, Cote d’Ivoire, Equatorial Guinea, Gabon, Guinea, Guinea Bissau, Mali, Mauritania, Niger, Senegal and Togo do not provide legislative exceptions for regulatory review purposes.
Compulsory licensing	Algeria, Angola, Botswana, Burundi, Cabo Verde, Democratic Republic of Congo, Eswatini, Ethiopia, The Gambia, Ghana, Kenya, Lesotho, Liberia, Malawi, Mozambique, Namibia, Nigeria, Rwanda, Sierra Leone, Sao Tome & Principe, Seychelles, South Africa, South Sudan, Tanzania, Uganda, Zambia, Zimbabwe, and OAPI countries namely Benin, Burkina Faso, Cameroon, Central African Republic, Chad, Comoros, Congo, Cote d’Ivoire, Equatorial Guinea, Gabon, Guinea, Guinea Bissau, Mali, Mauritania, Mauritius, Niger, Senegal and Togo.	
Exhaustion of rights and parallel i**m**portation	International exhaustion – Botswana, Burundi, Ghana, Kenya, Liberia, Mauritius, Namibia, Seychelles, Sierra Leone, South Africa, Zambia, Zimbabwe Regional Exhaustion – Benin, Burkina Faso, Cameroon, Central African Republic, Chad, Congo Republic, Cote d’Ivoire, Equatorial Guinea, Gabon, Guinea, Guinea-Bissau, Mali, Mauritania, Niger, Senegal, Togo – essentially all OAPI countries. National exhaustion – Eswatini, The Gambia, Lesotho, Rwanda, Madagascar, Mozambique, and Tanzania, Nigeria, Sao Tome & Principe, South Sudan, Uganda Algeria – legislation^a^ does not specify applicable level of exhaustion whether national or international	
Patent term extension	Angola^b^, Zimbabwe^c^	
Limits on test data protection	Uganda	
Transition periods for LDCs and exemption of pharmaceutical products from patentable subject matter	Angola, Burundi, Madagascar, Liberia, Rwanda, Uganda have excluded pharmaceutical products from patentability criteria	OAPI countries –Benin, Burkina Faso, Cameroon, Central African Republic, Chad, Comoros, Congo, Cote d’Ivoire, Equatorial Guinea, Gabon, Guinea, Guinea Bissau, Mali, Mauritania, Niger, Senegal and Togo, do not exempt pharmaceutical products from patentable subject matter.
Enactment of a patent opposition system	Countries with a pre-grant opposition system: - Botswana, Burundi, Cabo Verde, Lesotho, Liberia, Malawi, Mozambique, Namibia, Seychelles, Uganda, Zambia, Zimbabwe Countries with a post-grant opposition system: - Botswana, Comoros, DRC, Eswatini, Gambia, Ghana, Kenya, Lesotho, Liberia, Malawi, Mauritius, Mozambique, Namibia, Rwanda, Sao Tome & Principe, Seychelles, South Africa, South Sudan, Tanzania, Uganda, Zambia, Zimbabwe	OAPI countries –Benin, Burkina Faso, Cameroon, Central African Republic, Chad, Comoros, Congo, Cote d’Ivoire, Equatorial Guinea, Gabon, Guinea, Guinea Bissau, Mali, Mauritania, Niger, Senegal and Togo do not have patent opposition legislation.

^a^Article 12(2) of Ordinance No. 03–07 of July 19, 2003 on Patents

^b^Article 6 (2) of Law No. 3/92 of February 28, 1992 on Industrial Property states that once the patent validity period of 15 years expires, the subject of the patent shall fall into the public domain

^c^Section 24B (2) of the Zimbabwe Patents Act, 2002 states that where test batches of a patented product (through Bolar exception)… the term of a patented product shall not be extended.

## Appendix 4

**Table 5:** Summary of application of TRIPS flexibilities by African Region countries

Country	Date	Flexibility	Product	Patent filed/granted	Disease	Executed	Reason if not executed
Angola	2005	Par7	All medicines	Yes	All	Yes	
Benin	2004	Par7	ARVs	Yes	HIV/AIDS	Yes	
Benin	2007	Par7	ARVs	Yes	HIV/AIDS	Yes	
Benin	2009	Par7	ARVs	Yes	HIV/AIDS	Yes	
Burkina Faso	2005	Par7	ARVs +	Yes	HIV/AIDS	Yes	
Burundi	2005	Par7	ARVs +	Yes	HIV/AIDS	Yes	
Cameroon	2005	Art 31	NVP, 3TC, 3TC/AZT	Yes	HIV/AIDS	No	No response
Cape Verde	2004	Par7	All medicines	Unknown	All	Yes	
CAR	2004	Par7	ARVs +	Yes	HIV/AIDS	Yes	
CAR	2005	Par7	ARVs +	Yes	HIV/AIDS	Yes	
Chad	2005	Par7	ARVs	Yes	HIV/AIDS	Yes	
Chad	2007	Par7	All medicines	Yes	All	Yes	
Comoros	2007	Par7	ARVs +	Unknown	HIV/AIDS	Yes	
Congo	2007	Art 31	ARVs	Yes	HIV/AIDS	Yes	
Congo	2014	Art 31	ARVs	Yes	HIV/AIDS	Yes	
Congo	2005	Art 31	ARVs +	Yes	HIV/AIDS	Yes	
DRC	2005	Par7	ARVs	No	HIV/AIDS	Yes	
Eritrea	2005	Par7	ARVs	No	HIV/AIDS	Yes	
Ethiopia	2004	Art 31	ARVs	No	HIV/AIDS	Yes	
Gabon	2005	Art 31	ARVs	Yes	HIV/AIDS	Yes	
Gabon	2006	Art 31	ARVs	Yes	HIV/AIDS	Yes	
Gabon	2013	Art 31	ARVs	Yes	HIV/AIDS	Yes	
Gambia	2004	Par7	ARVs	Yes	HIV/AIDS	Yes	
Gambia	2007	Par7	All medicines	Yes	All	Yes	
Ghana	2005	Art 31	ARVs	Yes	HIV/AIDS	Yes	
Guinea	2004	Art 31	ARVs	Yes	HIV/AIDS	Yes	
Guinea	2005	Par7	ARVs	Yes	HIV/AIDS	Yes	
Guinea Bissau	2005	Par7	ARVs	Yes	HIV/AIDS	Yes	
Guinea Equatorial	2009	Art 31	ARVs	Yes	HIV/AIDS	Yes	
Ivory Coast	2004	Art 31	ARVs	Yes	HIV/AIDS	Yes	
Ivory Coast	2007	Art 31	3TC, 3TC/AZT, 3TC/AZT/NVP, 3TC/D4T, 3TC/D4T/NVP, DDI, EFV, IDV	Yes	HIV/AIDS	Yes	
Ivory Coast	2007	Art 31	ARVs	Yes	HIV/AIDS	Yes	
Kenya	2002	Par5d	Generics	Yes	All	Yes	
Kenya	2004	Art 31	3TC	Yes	HIV/AIDS	No	Voluntary licence
Kenya	2004	Art 31	3TC/AZT	Yes	HIV/AIDS	No	Voluntary licence
Kenya	2004	Art 31	AZT	Yes	HIV/AIDS	No	Voluntary licence
Kenya	2004	Art 31	NVP	Yes	HIV/AIDS	No	Voluntary licence
Lesotho	2004	Par7	ARVs	Yes	HIV/AIDS	Yes	
Lesotho	2006	Par7	All medicines	Yes	All	Yes	
Liberia	2005	Art 31	ARVs	No	HIV/AIDS	Yes	
Malawi	2004	Par7	All medicines	Yes	All	Yes	
Malawi	2005	Par7	ARVs	Yes	HIV/AIDS	Yes	
Mauritania	2004	Par7	ARVs	Yes	HIV/AIDS	Yes	
Mozambique	2004	Art 31	3TC/D4T/NVP	No	HIV/AIDS	Yes	
Mozambique	2005	Art 31	EFV	No	HIV/AIDS	Yes	
Mozambique	2005	Par7	ARVs	Yes	HIV/AIDS	Yes	
Niger	2004	Par7	All medicines	Yes	All	Yes	
Niger	2008	Par7	ARVs	Yes	HIV/AIDS +	Yes	
Rwanda	2007	Par7	3TC/AZT/NVP	No	HIV/AIDS	Yes	
Rwanda	2007	Par7	All medicines	Yes	All	Yes	
Senegal	2006	Par7	ARVs +	Yes	HIV/AIDS +	Yes	
Sierra Leone	2009	Par7	IDV, LPV/r, NVP, TDF	No	HIV/AIDS	Yes	
Sierra Leone	2009	Par7	DDI, IDV, LPV/r	No	HIV/AIDS	Yes	
South Africa	2003	Art 31	AZT, 3TC, AZT/3TC, NVP	Yes	HIV/AIDS	No	Voluntary licence
South Sudan	2007	Par7	All medicines	Yes	HIV/AIDS	Yes	
Eswatini	2005	Art 31	NVP, AZT	Yes	HIV/AIDS	Yes	
São Tomé and Príncipe	2006	Art 31	ARVs	No	HIV/AIDS	Yes	
Tanzania	2008	Par7	All medicines	Yes	All	Yes	
Togo	2004	Par7	All medicines	Yes	All	Yes	
Togo	2008	Par7	TDF/3TC	Yes	HIV/AIDS	Yes	
Togo	2009	Par7	EFV, NVP, 3TC/AZT	Yes	HIV/AIDS	Yes	
Uganda	2006	Par7	3TC/D4T/NVP	Yes	HIV/AIDS	Yes	
Zambia	2004	Art 31	3TC/D4T/NVP	Yes	HIV/AIDS	Yes	
Zambia	2004	Par7	ARVs +	Yes	HIV/AIDS +	Yes	
Zambia	2006	Par7	ARVs +	Yes	HIV/AIDS +	Yes	
Zimbabwe	2002	Art 31	ARVs +	Yes	HIV/AIDS +	Yes	
Zimbabwe	2003	Art 31	ARVs +	Yes	HIV/AIDS +	Yes	
Zimbabwe	2004	Art 31	ARVs	Yes	HIV/AIDS	Yes	
Zimbabwe	2005	Art 31	ARVs +	Yes	HIV/AIDS	Yes	

## Abbreviations

3TC: Lamivudine
AIDS: Acquired Immune Deficiency Syndrome
ARIPO: African Regional Intellectual Property Organization
ARV: Antiretrovirals
AZT: Azidothymidine
COVID-19: Corona Virus Disease of 2019
D4T: Stavudine
DG: Director General
DNA: Deoxyribonucleic Acid
DNDi: Drugs for Neglected Diseases Initiative
EFV: Efavirenz
EMRO: Eastern Mediterranean Region
EVD: Ebola Disease Virus
GSK: GlaxoSmithKline
GSPOA-PHI: Global Strategy and Plan of Action on Public Health, Innovation and Intellectual Property
HIV: Human Immuno Deficiency Syndrome
IP: Intellectual Property
IPC: International Patent Classification
IPP: Intellectual Property Protection
IPR: Intellectual Property Rights
LDC: Least Developed Countries
MS: Microsoft
NVP: Nevirapine
OAPI: Organisation Africaine de la Propriete Intellectuelle
PDP: Product Development Partnership
R&D: Research and Development
RNA: Ribonucleic Acid
TDF: Tenofovir Disoproxil Fumarate
TRIPS: Agreement on Trade-Related Aspects of Intellectual Property
USA: United States of America
US FDA: United States Food and Drug Administration
WHA: World Health Assembly
WHO: World Health Organization
WIPO: World Intellectual Property Organization
WTO: World Trade Organization

## Footnotes

1 In 2012 the WHO Consultative Expert Working Group on R&D: Financing and Coordination (CEWG) recommended that governments begin negotiations over a global medical R&D convention to address the problems of the current medical R&D system in a systematic way. See full report on https://apps.who.int/iris/bitsream/handle/10665/254706/9789241503457-eng.pdf;sessionid=DD5FA93B35229C40C7D989BBF9B8E6F8?sequence=1(accessed on 19 June 2020)

2 In January 2017 Article 31*bis* came into force thus amending Article 31(f) and (h) of the TRIPS Agreement to allow countries producing generic medicines under compulsory licensing to export those medicines to LDCs lacking manufacturing capacity

3 These are Algeria, Angola, Benin, Botswana, Burkina Faso, Burundi, Cabo Verde, Cameroon, Central African Republic, Chad, Comoros, Congo, Cote d’Ivoire, Democratic Republic of Congo, Equatorial Guinea, Eritrea, Eswatini, Ethiopia, Gabon, Gambia, Ghana, Guinea, Guinea Bissau, Kenya, Lesotho, Liberia, Madagascar, Malawi, Mali, Mauritania, Mauritius, Mozambique, Namibia, Niger, Nigeria, Rwanda, Sao Tome & Principe, Senegal, Seychelles, Sierra Leone, South Africa, South Sudan, Togo, Uganda, United Republic of Tanzania, Zambia, Zimbabwe

4 Was first identified in December 2019 as a respiratory illness caused by a novel coronavirus, Severe Acute Respiratory Syndrome Coronavirus 2 (SARS-Cov-2). See generally Helmy YA et.al. The COVID-19 pandemic: a comprehensive review of taxonomy, genetics, epidemiology, diagnosis, treatment, and control. J Clin Med 2020; 9 (4) E1225

5 USFDA. Coronavirus (COVID-19) update: FDA issues Emergency Authorization for Potential COVID-19 Treatment. https://www.fda.gov/news-events/press-announcements/coronavirus-covid-19-update-fda-issues-emergency-use-authorization-potential-covid-19-treatment (Accessed on 27 July 2020)

6 Gilead Sciences. https://www.gilead.com/news-and-press/company-statements/gilead-sciences-statement-on-request-to-rescind-remdesivir-orphan-drug-designation (accessed on 28 July 2020)

7 Article 33

8 It is noted that some low and middle income countries such as Albania, Argentina, Bolivia, Brazil, Colombia, Ecuador, Iran, Iraq, Jordan, Malaysia, Kosovo, Lebanon, Mexico, Montenegro, Paraguay, among others, are not covered under these non- exclusive license agreements

9 WHA 49.14

10 For example WHA56.27 (2003) on Intellectual Property Rights, Innovation and Public Health; WHA59.24 (2006) on Public Health, Innovation, Essential Health Research and Intellectual Property Rights: Towards a Global Strategy and Plan of Action; WHA59.26 (2006) on International Trade and Health; WHA60.30(2007) on Public Health, Innovation and Intellectual Property; WHA 62.16 (2009) on Global Strategy & Plan of Action on Public Health, Innovation and Intellectual Property; WHA65.22 (2012) on Follow up to the Report of the Consultative Expert Working Group on Research and Development: Financing and Coordination

11 CIPIH Report (WHO, 2006)

12 Through resolution WHA62.16

13 Article 4 (1)

14 Cluster A on Technical Assistance and Capacity Building

15 https://www.wipo.int/directory/en/

16 Algeria, Botswana, Burundi, Cameroon, Central African Republic, Chad, Congo, Democratic Republic of Congo, Eswatini, Gambia, Kenya, Lesotho, Liberia, Malawi, Mali, Mauritius, Mozambique, Namibia, Nigeria, Rwanda, Sao Tome and Principle, Seychelles, Sierra Leone and Uganda.

17 WIPO technical assistance may also be in the form of support to attend meetings, workshops and/or training and also for buying equipment.

18 https://www.aripo.org/

19 http://www.oapi.int/index.php/fr/

20 http://wto.org/english/thewto_e/min01_e/mindecl_trips_e.htm (Accessed on 19 June 2020)

21 Which states that countries ‘…shall be free to determine the appropriate method of implementing the provisions of this Agreement within their own legal system and practice’

22 Which makes specific mention of public health protection and states that “Members may … adopt measures necessary to protect public health and nutrition, and to promote the public interest in sectors of vital importance to their socio-economic and technological development, provided that such measures are consistent with the provisions of this Agreement.”

23 Available on WIPO Lex at https://www.wipo.int/wipolex/en/index.html

24 https://medicineslawandpolicy.org/

25 At present only 12 WHO African Region Member States namely, Algeria, Burkina Faso, Congo, Gambia, Kenya, Madagascar, Sao Tome and Principe, South Africa, Uganda, Tanzania, Zambia and Zimbabwe have responded to the online WIPO questionnaire.

26 It states ‘the TRIPS Agreement does not and should not prevent Members from taking measures to protect public health’ and that it ‘can and should be interpreted and implemented in a manner supportive of WTO Member’s rights to protect public health and, in particular to promote access to medicines for all’

27 WTO, IPC/C/64

28 WTO, IP/C/73

29 WTO, WT/L/971

30 See n 44

31 Botswana, Eswatini, the Gambia, Ghana, Kenya, Lesotho, Liberia, Malawi, Mauritius, Mozambique, Namibia, Rwanda, Sao Tome and Principe, Sierra Leone, Uganda, United Republic of Tanzania, Zambia and Zimbabwe (Somalia and Sudan are the other 2 members and are members of the WHO Eastern Mediterranean Region)

32 Discussions with study key informant, April 2019

33 Article 2 (xii – xv) defines what a national, designated, elected and or receiving patent office are respectively.

34 Which defines non-patentable subject matter

35 Which provides for patent application requirements, including the documents required

36 which provides that a patent application shall be restricted to a single principal subject and that it shall have a title that describes in a precise and succinct manner the purpose of the invention

37 https://www.wipo.int/wipolex/en/index.html

38 Section 17 (1) (j) Industrial Property Act No. 11 of 2017

39 Article 18.5 of Law No. 31/2009 on the Protection of Intellectual Property

40 Section 17 (e) Patents Act No. 40 of 2016

41 Algeria, Botswana, Burundi, Cabo Verde, Democratic Republic of Congo, Eswatini, Ethiopia, Ghana, Kenya, Lesotho, Liberia, Madagascar, Mauritius, Mozambique, Namibia, Rwanda, Sao Tome & Principe, Seychelles, Sierra Leone, Tanzania, Uganda

42 Botswana, Cabo Verde, Kenya, Liberia, Namibia, Rwanda, Sao Tome & Principe, Seychelles, South Africa, Uganda, Zambia, Zimbabwe

43 Article 6 (2) of Law No. 3/92 of February 28, 1992 on Industrial Property states that once the patent validity period of 15 years expires, the subject of the patent shall fall into the public domain

44 Section 24B (2) of the Zimbabwe Patents Act, 2002 states that where test batches of a patented product (through Bolar exception)… the term of a patented product shall not be extended.

45 Angola, Benin, Burkina Faso, Burundi, Central African Republic, Chad, Comoros, Democratic Republic of Congo, Eritrea, Ethiopia, Gambia, Guinea, Guinea Bissau, Lesotho, Liberia, Madagascar, Malawi, Mali, Mauritania, Mozambique, Niger, Rwanda, Sao Tome & Principe, Senegal, Sierra Leone, South Sudan, Togo, Uganda, Tanzania, Zambia

46 Article 4 (d) of Law No. 3/92 of February 28, 1992 on Industrial Property

47 Section 1 (8) (1) (v) Ordinance No. 89–019 of July 31, 1989 establishing Agreements for the Protection of Industrial Property

48 Section 13 (2) (b) Liberia Intellectual Property Act 2016

49 Article 18 (8) Rwanda Law on Protection of Intellectual Property 2009

50 Section 8 (3) (f) Uganda Industrial Property Act 2014

51 Article 17 of Law No 1/13 of July 28, 2009 relating to Industrial Property in Burundi

52 Found on http://tripsflexibilities.medicineslawandpolicy.org/

53 At present only 12 WHO African Region Member States namely, Algeria, Burkina Faso, Congo, Gambia, Kenya, Madagascar, Sao Tome and Principe, South Africa, Uganda, Tanzania, Zambia and Zimbabwe have responded to the online WIPO questionnaire.

54 Available on https://www.wipo.int/scp/en/exceptions/

55 This provision states that compulsory licensing shall be authorized predominantly for the supply of the domestic market of the country authorizing it

56 Which states that ‘the right holder shall be paid adequate remuneration in the circumstances of each case, taking into account the economic value of the authorization’

57 See a supporting view in Correa, C.M. Will the Amendment to the TRIPS Agreement Enhance Access to Medicines? South Centre Policy Brief No. 57 January 2019

58 Namely Benin, Burkina Faso, Central African Republic, Chad, Gambia, Guinea, Guinea-Bissau, Lesotho, Liberia, Malawi, Mali, Mauritania, Mozambique, Niger, Rwanda, Senegal, Sierra Leone, Togo, Uganda, Tanzania and Zambia

59 Article 70.8 provides “Where a Member does not make available as of the date of entry into force of the WTO Agreement patent protection for pharmaceutical and agricultural chemical products commensurate with its obligations under Article 27, that member shall:

- notwithstanding the provisions of Part IV, provide as from the date of entry into force of the WTO Agreement a means by which applications for patents for such inventions can be filed;
- apply to these applications, as of the date of application of this Agreement, the criteria for patentability as laid down in this Agreement as if those criteria were being applied on the date of filing in that Member or, where priority is available and claimed, the priority date of the application; and
- provide patent protection in accordance with this Agreement as from the grant of the patent and for the remainder of the patent term, counted from the filing date in accordance with Article 33 of this Agreement, for those of these applications that meet the criteria for protection referred to in subparagraph (b).

60 Article 70.9 states “Where a product is the subject of a patent application in a Member in accordance with paragraph 8 (a), exclusive marketing rights shall be granted, notwithstanding the provisions of Part IV, for the period of 5 years after obtaining marketing approval in that Member or until a product patent is granted or rejected in that Member…”

61 WTO, IP/C/73
